# Serum Iron and Its Relationship with Hematologic Parameters in Healthy Female Alpacas at Two Different Ages

**DOI:** 10.1007/s12011-025-04757-0

**Published:** 2025-07-18

**Authors:** Matthias Gerhard Wagener, Max Kornblum, Frederik Kiene, Martin Ganter, Ulrike Teichmann

**Affiliations:** 1 Clinic for Swine and Small Ruminants, Forensic Medicine and Ambulatory Service, University of Veterinary Medicine Hannover, Foundation, Hannover, Germany; 2 Max Planck Institute for Multidisciplinary Sciences, Göttingen, Germany

**Keywords:** South American camelids, Alpaca, Iron, Hematology, Anemia

## Abstract

Iron is an important trace element whose deficiency is associated with anemia, which is a common condition in South American camelids. However, there are only few data available on the physiologic relationships between serum iron and hematologic findings in alpacas. In 81 laboratory diagnostic datasets from 21 clinically healthy alpacas collected over a nine-year period, we recently reported significant positive correlations between iron and hemoglobin (Hb), hematocrit (HCT), mean corpuscular volume (MCV), mean corpuscular hemoglobin (MCH), and eosinophils, and significant negative correlations between iron and mean corpuscular hemoglobin concentration (MCHC), platelets, and neutrophils. For 11 of the animals, datasets were available both at the age of 1 year (young) and at an older age (4–7 years: mature). For young animals, significant positive correlations were found between iron and MCV, MCH, and eosinophils, and significant negative correlations between iron and white blood cells (WBC), red blood cells (RBC), platelets, and neutrophils. In contrast, in mature animals, significant positive correlations were found between iron and monocytes as well as eosinophils. A two-way ANOVA of these 11 animals examining the effects of age, iron, and age*iron revealed significant effects of age on eosinophils, of iron on RBC and MCH, and of age*iron on WBC, RBC, HCT, and lymphocyte-to-monocyte ratio (LMR). Our data show that both age and serum iron have an effect on hematologic parameters in alpacas. Especially in young animals there is a close association between iron and MCH and MCV and neutrophils.

**Supplementary Information:**

The online version contains supplementary material available at 10.1007/s12011-025-04757-0.

## Introduction

Iron is an essential trace element for animals and humans where most of the iron can be found in red blood cells [1]. As iron is an important component of hemoglobin, iron deficiency can lead to inadequate erythropoiesis, resulting in iron deficiency anemia [2]. Anemia is a common condition in South American camelids (SACs; alpacas and llamas) [3, 4]. In a retrospective analysis of data from 300 SACs presented to our clinic, 49% of alpacas and 55% of llamas were found to have a packed cell volume (PCV; defined as the percentage of erythrocytes in the blood measured by centrifugation in a capillary [5]) below the reference intervals [6]. As SACs are stoic animals, a poor health condition can often go unrecognized for a long time [7]. Some of those emaciated animals reveal a significantly decreased PCV of less than 0.10 L/L, which is a life-threatening situation for the affected animal [8]. The most common cause of a severe decrease in PCV is usually an infection with *Haemonchus contortus*, a gastrointestinal hemophagous helminth that can cause dramatic blood loss in SACs [9, 10]. Other conditions that were associated in the literature with anemia in SACs include infections with *Candidatus* Mycoplasma haemolamae [11], intoxications [12], or trace element deficiencies [13–15]. However, the underlying cause of anemia is not diagnosed in every case, particularly when the anemia is only moderate [3].

In humans, anemia is mostly caused by iron deficiency due to increased iron requirements, low iron intake, decreased intestinal iron absorption, chronic blood loss, or multiple causes [16, 17]. In veterinary medicine, iron deficiency anemia is also a relevant issue [5, 18, 19]. In contrast to humans, the relevance varies depending on the species, as the amount of ingested iron varies depending on the diet [1]. Nutritive iron deficiency in suckling piglets and chronic blood loss due to hemonchosis in SACs are examples of different causes of iron deficiency in farm animals [8, 9, 20]. In addition, any chronic inflammation can be a reason for anemia, as hepcidin induces a drop in available serum iron, which leads to reduced hemoglobin synthesis [21].

Although a close association between the hematological parameters and iron is well known from other species [5], testing for trace element status in alpacas and llamas often plays a minor role in routine veterinary practice. This might be due to the fact that only little information on the role of trace elements and anemia in SACs is available in the literature to date.

Weiser et al. [22] provided first data on the iron content and erythrocytic indices of healthy llamas in 1992, Morin and colleagues subsequently investigated iron deficiency and its impact on hematologic parameters in llamas [15, 23]. We have previously documented a case of severe anemia in a llama due to hemonchosis with associated iron deficiency [8]. Even though there is already little information on this topic in llamas, there is a lack of data on the relationship between iron and hematologic parameters in alpacas.

In a recently published study, we investigated age-related changes in hematologic parameters in alpacas [24]. We found significant age-dependencies for hemoglobin (Hb), mean corpuscular volume (MCV), mean corpuscular hemoglobin (MCH), hematocrit (HCT; defined as the calculated result from MCV and RBC in a hematological analyzer [5]), neutrophils, lymphocytes, platelet-to-lymphocyte ratio (PLR), neutrophil-to-lymphocyte ratio (NLR), and lymphocyte-to-monocyte ratio (LMR). For this purpose, we analyzed laboratory diagnostic data from 21 healthy female alpacas from an experimental herd over a period of nine years retrospectively. In addition to hematology, serum samples, which were taken simultaneously, were analyzed for associations of serum parameters and age [25]. However, in contrast to the hematologic parameters, no significant correlation were detected for iron and age of the animals (*r* = 0.16; *p* = 0.16) [25].

In this follow-up study, we want to show the relationship between hematologic parameters and serum iron concentrations in healthy alpacas at two different ages. On the one hand we assume relationships between serum iron and the parameters of the red blood count, as serum iron is associated with hemoglobin and red blood cell synthesis. On the other hand we investigate relationships between serum iron and the parameters of the white blood count, as chronic inflammation can be associated with reduced serum iron.

## Material and Methods

A previous retrospective study was carried out based on laboratory diagnostic data from an alpaca herd kept for the production of nanobodies. A total of 87 hematologic records from 21 female alpacas were identified over a nine-year period (2013–2022). Based on the inclusion criteria, the animals were considered clinically healthy and were not used in an immunization study at the time of sample collection. A detailed overview of the history and the management of the herd as well as inclusion and exclusion criteria for the study are provided in the previous paper [24]. For 81 of the hematologic examinations, iron was measured in a simultaneously taken serum sample. The samples were sent to an external commercial laboratory (SYNLAB.vet GmbH, Augsburg, Germany) which was accredited by the “Deutsche Akkreditierungsstelle GmbH”, certificate D-PL-14016–01-00 in accordance with DIN EN ISO/IEC 17025:2018 in 2020.

Detailed information on sampling and the measurement of the hematologic and serum parameters in those animals have been reported previously [24, 25].

As the previous studies showed age-related changes in the hematologic parameters, albeit no changes in the serum iron content over the course of age were detectable, we investigated the relationship between iron [µmol/L] and the hematologic parameters (white blood count (WBC) [G/L], RBC [T/L], hemoglobin (Hb) [g/L], HCT [L/L], MCV [fL], MCH [pg], MCHC [g/L], platelets [G/L], neutrophils [% and G/L], lymphocytes [% and G/L], monocytes [% and G/L], eosinophils [% and G/L], basophils [% and G/L], NLR, PLR, and LMR) statistically. Analysis was performed using SAS Enterprise Guide, version 7.1 (SAS Institute Inc., Cary, NC, USA) and divided into three different evaluations:

1. Correlation analysis between serum iron and the hematologic parameters for all available sampling points at different ages (“all”: group “A”). For data where the Shapiro–Wilk test revealed a *p-* value ≥ 0.05, the Pearson correlation coefficient was used; for data where the Shapiro–Wilk test revealed a *p-* value < 0.05, the Spearman correlation coefficient was used. Significant correlations were interpreted as follows: r = 0–0.10: negligible correlation; r = 0.10–0.39: weak correlation; r = 0.40–0.69: moderate correlation; r = 0.70–0.89: strong correlation; r = 0.90–1.00: very strong correlation [26].
2. Correlation analysis between serum iron and the hematologic parameters of 11 animals in which the values were collected at two times at the age of 1 year (“young”: group “Y”) as well as at a later stage (4 years: *n* = 8; 5 years: *n* = 1; 6 years: *n* = 1; 7 years: *n* = 1; “mature”: group “M”) in separate calculations. For group M, only the earliest sample, taken after the animals had reached the age of 4 years, was included, as the samples were taken at irregular intervals. Correlation analysis was performed according to evaluation 1. The classification into groups Y and M was chosen because South American camelids grow until the age of two to three years [27, 28]. Maturity should therefore be assumed from the age of 4 years onwards.
3. A two-way analysis of variance (ANOVA) in which the hematologic parameters were analyzed in relation to the factors iron, age, and iron*age. Age was divided into both groups Y and M as described in evaluation 2. Iron was divided into the two groups “iron high” (IH) and “iron low” (IL). The median and mean of the total of 81 iron values were 25.6 µmol/L and 26.1 µmol/L, respectively [25]. Iron concentrations ≥ 26.0 µmol/L were classified as IH and iron concentrations < 26.0 µmol/L as IL. Serum iron of group Y and group M was statistically compared using a paired t-test.

Basophils were only included in evaluation 1 because no basophils were detected in the records of the 11 animals included in evaluations 2 and 3 at both evaluated time points. A detailed overview of the individual animals and the respective age groups from which samples were taken for groups A, Y and M is provided in Supplementary Table 1.

## Results

Correlation analysis for serum iron and the hematologic parameters for all 81 records revealed moderate positive correlations for serum iron and HCT, MCV, and MCH, weak positive correlations for serum iron and hemoglobin and eosinophils, as well as weak negative correlations for serum iron and MCHC, platelets, and neutrophils. Correlation analysis of serum iron and the other hematologic parameters revealed no significant results (Table 1). However, as previous studies showed that Hb, HCT, MCV, MCH, neutrophils, PLR, and NLR were correlated positively, and lymphocytes and LMR were correlated negatively with age [24], while serum iron did not show a significant correlation with the age of the animals [25], we assumed that the correlations found here could also be an effect of age. Therefore, we examined the correlations between serum iron and hematologic parameters separately for 11 animals at the time points Y and M (Table 1). At one year of age (Y), the animals showed very strong significant positive correlations for serum iron and MCV as well as MCH and a moderate positive correlation for serum iron and the relative number (percentage) of eosinophils. Nonetheless, at the same time, a strong negative correlation between serum iron and the absolute number of neutrophils as well as moderate negative correlations between serum iron and WBC, RBC, and platelets were observed. At a more advanced age (M), only strong positive correlations between serum iron and the absolute numbers of monocytes and eosinophils could be observed, whereas correlation analysis of the other hematologic parameters and serum iron revealed no significant results (Table 1).

**Table 1:** Results of the correlation analyses of serum iron [µmol/L] and age of the animals during sampling [days] and the hematologic parameters for all blood samples for which both a hematologic examination and a measurement of serum iron were available (*n* = 81 records from 21 female alpacas; “all”; A) as well as for 11 animals for which examinations were performed at 1 year of age (“young”; Y) and repeated at a later time (between 4 and 7 years of age; “mature”; M). The lymphocyte-to-monocyte ratio (LMR) has a lower n than the other parameters because in these cases where no monocytes were present, no LMR could be calculated. Significant results are shown in bold. A detailed overview of the individual animals and sampling times can be found in Supplementary Table 1. Detailed descriptive statistics for these groups are shown in Supplementary Tables 2, 3, 4

	All (A)			Young (Y)			Mature (M)		
Parameter	*n* =	*r* =	*p* =	*n* =	*r* =	*p* =	*n* =	*r* =	*p* =
Age [days]	81	0.16	0.16	11	0.32	0.34	11	−0.15	0.67
WBC [G/L]	81	−0.10	0.39	**11**	**−0.62**	** < 0.05**	11	0.53	0.09
RBC [T/L]	81	−0.01	0.95	**11**	**−0.64**	** < 0.05**	11	0.21	0.53
Hb [g/L]	**81**	**0.38**	** < 0.001**	11	0.03	0.94	11	0.47	0.15
HCT [L/L]	**81**	**0.41**	** < 0.001**	11	0.16	0.63	11	0.43	0.19
MCV [fL]	**81**	**0.56**	** < 0.001**	**11**	**0.95**	** < 0.001**	11	0.32	0.33
MCH [pg]	**81**	**0.45**	** < 0.001**	**11**	**0.91**	** < 0.001**	11	0.32	0.33
MCHC [g/L]	**81**	**−0.23**	** < 0.05**	11	−0.26	0.45	11	−0.17	0.62
Platelets [G/L]	**81**	**−0.25**	** < 0.05**	**11**	**−0.62**	** < 0.05**	11	−0.39	0.23
Neutrophils [%]	**81**	**−0.31**	** < 0.01**	11	−0.39	0.24	11	−0.54	0.08
Lymphocytes [%]	81	0.01	0.95	11	−0.07	0.83	11	−0.09	0.80
Monocytes [%]	81	0.04	0.71	11	−0.15	0.65	11	0.54	0.08
Eosinophils [%]	**81**	**0.28**	** < 0.05**	**11**	**0.66**	** < 0.05**	11	0.49	0.12
Basophils [%]	81	0.10	0.40	11	n.d	n.d	11	n.d	n.d
Neutrophils [G/L]	**81**	**−0.25**	** < 0.05**	**11**	**−0.73**	** < 0.05**	11	0.14	0.67
Lymphocytes [G/L]	81	−0.04	0.75	11	−0.55	0.08	11	0.55	0.08
Monocytes [G/L]	81	−0.02	0.89	11	−0.36	0.28	**11**	**0.72**	** < 0.05**
Eosinophils [G/L]	**81**	**0.23**	** < 0.05**	11	0.43	0.19	**11**	**0.74**	** < 0.01**
Basophils [G/L]	81	0.10	0.39	11	n.d	n.d	11	n.d	n.d
NLR	81	−0.13	0.26	11	−0.14	0.68	11	−0.23	0.49
PLR	81	−0.19	0.09	11	−0.22	0.51	11	−0.46	0.16
LMR	63	0.04	0.75	9	0.45	0.23	10	−0.57	0.09

*WBC*, White blood cell; *RBC*, Red blood cell; *Hb*, Hemoglobin; *HCT*, Hematocrit; *MCV*, Mean corpuscular volume; *MCH*, Mean corpuscular hemoglobin; *MCHC*, Mean corpuscular hemoglobin concentration; *NLR*, Neutrophil-to-lymphocyte ratio; *PLR*, Platelet-to-lymphocyte ratio; *n.d.*, not determined

The two-way ANOVA revealed a significant effect (*p* < 0.05) of age*iron on WBC, RBC, HCT, and LMR as well as a significant effect (*p* < 0.05) of iron on RBC and MCH. WBC, RBC, and HCT were higher in YIL than in YIH but lower in MIL than in MIH. LMR was lower in YIL than in YIH but higher in MIL than in MIH. MCH was lower in IL than in IH in both Y and M. Iron further revealed a statistical trend (*p* = 0.05) on MCV, which was also lower in IL than in IH in both Y and M. Age had a significant effect (*p* < 0.05) on the total number of eosinophils, which were lower in Y than M. The detailed results of the two-way ANOVA are displayed in Table 2; descriptive statistics can be found in Supplementary Table 1.

**Table 2:** Results of the two-way ANOVA. Hematologic parameters were analyzed in relation to the factors iron, age, and iron*age. Age was divided into both groups “young” (Y) and “mature” (M), iron into the groups “iron high” (IH; serum iron ≥ 26 µmol/L) and “iron low” (IL; serum iron < 26 µmol/L). Significant results are shown in bold. A detailed overview of the descriptive statistics for groups Y, M, IH, IL, YIL, YIH, MIL, and MIH can be found in Supplementary Tables 3 to 10

	age	iron	age*iron
Parameter	*p* =	*p* =	*p* =
WBC [G/L]	0.82	0.37	**0.04**
RBC [T/L]	0.13	**0.04**	**0.02**
Hb [g/L]	0.09	0.97	0.11
HCT [L/L]	0.07	0.66	**0.03**
MCV [fL]	0.20	0.05	0.43
MCH [pg]	0.78	**0.02**	0.67
MCHC [g/L]	0.28	0.27	0.82
Platelets [G/L]	0.22	0.40	0.74
Neutrophils [%]	0.77	0.55	0.33
Lymphocytes [%]	0.15	0.64	0.36
Monocytes [%]	0.96	0.74	0.09
Eosinophils [%]	**0.04**	0.17	0.88
Basophils [%]	n.d	n.d	n.d
Neutrophils [G/L]	0.64	0.95	0.11
Lymphocytes [G/L]	0.42	0.26	0.06
Monocytes [G/L]	0.90	0.96	0.10
Eosinophils [G/L]	0.06	0.13	0.32
Basophils [G/L]	n.d	n.d	n.d
NLR	0.18	0.79	0.50
PLR	0.56	0.58	0.15
LMR	0.42	0.78	**0.03**

*WBC*, White blood cell; *RBC*, Red blood cell; *Hb*, Hemoglobin; *HCT*, Hematocrit; *MCV*, Mean corpuscular volume; *MCH*, Mean corpuscular hemoglobin; *MCHC*, Mean corpuscular hemoglobin concentration; *NLR*, Neutrophil-to-lymphocyte ratio; *PLR*, Platelet-to-lymphocyte ratio; *LMR*, Lymphocyte-to-monocyte ratio;* n.d.*, not determined

## Discussion

In this retrospective study of the laboratory records of healthy female alpacas, we found significant correlations between serum iron and several hematologic parameters, which varied depending on the age of the animals. The most obvious effects were found for MCH and MCV, which correlated positively with serum iron in the dataset containing all samples and in the young animals (groups A and Y). This observation is most likely driven by the strong relationship between iron and these parameters, particularly in young animals. It is therefore recommended that alpacas with low MCH and MCV should be checked for iron deficiency, particularly if they are still juvenile. Similar observations were made in juvenile individuals of other species like pigs or cattle in which parenteral iron supplementation led to an increase in these parameters [29, 30]. One assumption explaining that no statistical correlation was found in group M could be that serum iron in group Y (22.9 ± 8.9 µmol/L) was numerically lower than in group M (26.4 ± 5.5 µmol/L). This difference, however, did not prove to be statistically significant (*p* = 0.22). This observation is consistent with previously reported findings from llamas; Smith et al. [31] studied iron in llamas of different ages and found that the mean iron concentration of the animals decreased from 192 µg/dL (34.4 µmol/L) after birth to 108 µg/dL (19.3 µmol/L) at 12 months of age. Thereafter, no further age-related fluctuations were observed. However, no comparable data is available for alpacas so far. For serum iron in alpacas there is information in studies on reference intervals, but the specified intervals in these studies sometimes have a wide range. Hengrave Burri et al. [32] indicated as reference interval for serum iron 19.1–36.1 µmol/L, Dawson et al. [33] indicated 13.4–34.3 µmol/L and Stanitznig et al. [34] 9.6–63.8 µmol/L. If the 81 records on iron from our study are compared with the reference intervals in this order, 9.9% (8/81), 3.7% (3/81) and 2.5% (2/81) were below these reference intervals, and 4.9% (4/81), 7.4% (6/81) and 0% (0/81) were above these reference intervals. As our data are obtained from a herd of clinically healthy animals, which were under close veterinary supervision, we assume that the range of records we determined (7.0–47.6 µmol/L) represents physiological fluctuations.

The results of evaluation 3 also suggested that MCH and MCV are influenced more by iron than by age. Weiser et al. [22] examined serum iron as well as RBC, MCV, Hb, and MCHC in 38 healthy llamas, but reported no test for correlations between serum iron and hematologic parameters. While RBC and Hb of those llamas were largely consistent with the alpacas examined here, there were deviations in MCV and MCHC, which were discussed in detail in the previously published study [24]. Morin et al. examined the hematologic findings of three llamas with iron deficiency (serum iron concentration: 20; 31; 60 µg/dL or 3.6; 5.6; 10.7 µmol/L) and found decreased PCV, Hb, MCV, and MCHC in all llamas, but decreased RBC only in the animal with the lowest serum iron [15]. In our evaluation, only in study 1 did Hb and HCT show significant positive correlations with serum iron. In a later study, Morin et al. [23] induced anemia by phlebotomy in two healthy llamas; in addition to decreased iron, hematology also showed decreased Hb, MCV, and MCHC levels. However, the findings normalized after the cessation of blood withdrawal without the need for iron supplementation [23].

The causes of iron deficiency in SACs are similar to those in ruminants and include inadequate dietary iron intake, for example, due to prolonged feeding on milk-based diets, or chronic blood loss [35, 36]. Especially prolonged external blood loss is a major problem. While iron can be recycled during internal blood loss, it is lost in external bleeding. If the anemia in this condition has a regenerative character in the early stages, it becomes increasingly non-regenerative thereafter [5]. This is particularly important in the case of hemonchosis, which represents external blood loss via the gastrointestinal tract and frequently occurs in SACs [9]. In addition, iron deficiencies were often observed in association with other deficiencies in SACs [37, 38]. In a llama presented to our clinic with severe anemia (PCV: 0.06 L/L) due to hemonchosis, we also found a severely reduced serum iron concentration (1.69 µmol/L) [8]. The complete recovery of the anemia in that case took several weeks despite parenteral iron supplementation.

MCH and MCV are also closely correlated with serum iron in other species such as sheep or cattle [36, 39]. However, lower serum iron does not necessarily appear to be associated with lower erythrocytic indices. In goats, Rastogi et al. found significantly lower serum iron and RBC in goats kept under nomadic conditions than in farm-raised goats. Nevertheless, the animals with lower serum iron had significantly higher MCH and MCV. PCV and Hb did not differ between the groups [40].

In addition to the erythrocyte parameters, we also found significant correlations for serum iron with the leukocytes and platelets. In terms of leukocytes, there were significant negative correlations between serum iron and neutrophils in groups A and Y.

Significant positive correlations between serum iron and eosinophils in groups A, Y, and M as well as significant positive correlations between serum iron and monocytes in group M were evident. These findings could be explained by the fact that in inflammatory conditions, iron decreases as part of the acute phase reaction [41, 42]. For humans and many other mammals it has been demonstrated that the expression of the acute phase protein hepcidin due to an inflammatory stimulus from the hepatocytes results in reduced duodenal uptake and reduced export of iron from the macrophages [43, 44]. It is assumed that this effect has a major influence on the development of anemia in chronic inflammation [21, 45]. The detected correlations support the assumption that these effects must also be taken into account in the treatment of anemic alpacas.

An association between serum iron concentration and inflammatory processes is also known from other species such as cattle, horses, dogs, or cats [41, 46, 47]. In inflammatory conditions such as mastitis or traumatic reticuloperitonitis in cattle, or gastroenteritis in dogs and cats, decreased serum iron concentrations were associated with increased neutrophils and especially band neutrophils [41, 47]. Although the alpacas in the study examined here were clinically healthy [24], there were comparable negative correlations between serum iron and neutrophil levels. Band neutrophils were not detected in the alpacas from the evaluated herd [24]. However, these comparisons with other species should be taken with caution, as there is a lack of information on these specific conditions in alpacas in the literature. Passler et al. [43] investigated inflammatory reactions in alpacas after lipopolysaccharide (LPS) administration. The LPS group in their study had significantly lower serum iron than the control group after 12 h, but no statistical differences in WBC and neutrophils were found between both groups. However, in parallel with decreasing serum iron, there was an increase in NLR in the LPS group, which can be used as a diagnostic marker for inflammatory processes and is calculated from neutrophils and lymphocytes. We did not find statistical correlations between NLR and serum iron in the animals we studied, probably due to the absence of inflammatory processes, since all observed individuals appeared clinically healthy. On the other hand, evaluation 3 showed an effect of age*iron on LMR. To date, there is insufficient information on LMR in veterinary medicine, for it is normally used as a tumor marker [48]. No clear trend can be derived from our findings, as different associations of LMR with serum iron were observed depending on age. Therefore, further studies on this parameter are necessary in alpacas. The positive correlation between serum iron and monocytes as well as eosinophils also remains unclear. Baydar & Dabak [41] did not find any comparable associations in the above-mentioned study on cattle. In addition, significant negative correlations between iron and platelets were found in groups A and Y. This is consistent with the findings of Roland et al., that showed that low iron levels in cattle can be associated with thrombocytosis [36]. The latter is also well known for humans and mice, where it has been recently shown that low iron promotes megakaryocytic commitment of megakaryocytic-erythroid progenitors [49].

The hemotropic bacterium *Candidatus* M. haemolamae, which is frequently found in SACs, has no relevant influence on the hematologic parameters according to current knowledge [50, 51]. In addition to iron, other deficiencies such as copper may be associated with anemia in SACs [13]. However, there are few data on this available.

Due to the retrospective character of the present study, several limitations must be considered, including the limited number of animals, the shipment of samples to an external laboratory, and the automated analysis methods used in the laboratory. It should also be noted that endoparasites and zinc deficiency, which are typical conditions in alpacas, have occurred in the herd studied in the past. In addition, some animals were used for immunizations or blood sampling at single points in time. Care was taken to ensure that only those data were used, where the animals were > 30 days after such an event was completed at the time of sampling, to ensure that the previous event had no influence on the parameters. However, if this was the case for a blood sample (*n* = 60), the actual interval was usually significantly longer than 30 days (median: 218 days, interquartile range: 119–337 days). Nevertheless, these limitations were already discussed in detail in our previously published study [24]. A further limitation is the selection of the animals in group M. As this was a retrospective study, the samples were not taken at defined points in time. The inclusion of three animals, from which the samples were taken at time point M when they were 5, 6, or 7 years old, was necessary to reach a total of 11 animals because it would not have been possible to carry out the two-way ANOVA with less animals. Maturity is reached at four years of age [27, 28], and alpacas can become up to twenty years old [52]. Therefore, we assume this did not cause significant bias. Even though the animals in this experimental herd were clinically healthy, they were not pathogen-free, but were exposed to environmental influences. The respective fluctuations in the parameters are to be understood in the physiological range. The relationships between age, iron and the hematological parameters determined in our evaluations, on the other hand, seem to represent physiological correlations, as they proved to be statistically significant. As there is little data on alpacas, we have also used comparisons with other species in our interpretation. However, these should be considered with caution and re-evaluated when more information on iron metabolism in alpacas is available.

In summary, serum iron and hematologic parameters are also closely related in alpacas. These findings can be useful in both directions: on the one hand, hematologic findings such as reduced MCH and MCV can indicate iron deficiency; on the other hand, iron deficiency can indicate anemia or inflammatory conditions. When interpreting our results, it should be noted that they are from clinically healthy animals, so we assume that the diagnosed correlations are physiological. These correlations may have a more pronounced effect in the case of pathological changes, such as a bacterial infection. Overall, the nutritional iron deficiency probably plays a rather subordinate role, as most cases of anemia, even in juvenile individuals, are related to blood loss caused by hemonchosis and gastric ulcers. Since an age-related correlation between serum iron concentration and erythrocyte quality can clearly be observed, parenteral iron supplementation in addition to elimination of the primary cause might be important for fast recovery during veterinary treatment of anemia in SACs, particularly in young animals.

## Supplementary Information

Below is the link to the electronic supplementary material.

Supplementary file1 (PDF 513 KB)

## Data Availability

Data is provided within the manuscript or supplementary information files.

## Abbreviations

A: All
Hb: Hemoglobin
HCT: Hematocrit
IH: Iron high
IL: Iron low
LMR: Lymphocyte-to-monocyte ratio
LPS: Lipopolysaccharide
M: Mature
MCH: Mean corpuscular hemoglobin
MCHC: Mean corpuscular hemoglobin concentration
MCV: Mean corpuscular volume
NLR: Neutrophil-to-lymphocyte ratio
PCV: Packed cell volume
PLR: Platelet-to-lymphocyte ratio
RBC: Red blood cell
SAC: South American camelid
WBC: White blood cell
Y: Young
