# Supplementary material for: Serum Iron and Its Relationship with Hematologic Parameters in Healthy Female Alpacas at Two Different Ages
Source: Biol Trace Elem Res. 2025 Jul 18;204(3):1434–41. doi: 10.1007/s12011-025-04757-0 (PMC12992375; doi:10.1007/s12011-025-04757-0)
Supplement: Supplementary file 1 — Supplementary file1 (PDF 513 KB) [file 12011_2025_4757_MOESM1_ESM.pdf]

Supplementary Tables

Supplementary Table 1:

Overview of the herd. The serial number, sex and date of birth of each animal are given. The numbers in the column Group “A” indicate the respective age of the animal for which a complete data set (hematological examination and serum iron) was available according to the inclusion criteria. For Group “Y”, the findings from age 1 year and for Group “M” the findings from age 4 (or 5, 6 or 7) years of the specified animals were evaluated.

Animal	Sex	Date of birth	Group "A"	Group "Y"	Group "M"
1	female	11.10.2019	1;2		
2	female	30.08.2018	1;2;3		
3	female	23.10.2010	4;5;7;9;11		
4	female	27.05.2012	1;7;8;10	1	7
5	female	21.08.2014	1;4;5;6;7	1	4
6	female	30.06.2018	1;3;4	1	4
7	female	27.02.2018	1;3,4	1	4
8	female	23.03.2018	1;2;3;4	1	4
9	female	04.06.2018	1;2;3;4	1	4
10	female	28.07.2013	<1;1;3;6;7;8	1	6
11	female	14.10.2013	2;3;5;6;7;8		
12	male	26.07.2016	excluded		
13	female	08.02.2018	1;2;3		
14	female	28.09.2017	3;4		
15	female	06.08.2011	1;3;5;8;10	1	5
16	female	10.09.2014	1;4;5;6;7	1	4
17	female	20.06.2019	1;3		
18	female	13.06.2011	2;5;7;8;9;11		
19	female	24.06.2012	1;4;7;9;10	1	4
20	female	30.06.2018	1;3;4	1	4
21	female	10.06.2019	1;2;3		
22	female	26.06.2020	<1;2		

Supplementary Table 2:

Descriptive results of the findings for group “A” (all) for the hematological parameters and for serum iron.

	n=	Mean	SD	Median	Min	Max	LQ	UQ
WBC [G/L]	81	13.2	3.7	12.6	7.0	22.9	10.4	15.6
RBC [T/L]	81	12.7	1.7	12.5	8.8	18.2	11.6	13.6
Hb [g/L]	81	135.3	14.6	136.0	108.0	174.0	125.0	146.0
HCT [L/L]	81	0.44	0.06	0.43	0.30	0.61	0.39	0.48
MCV [fL]	81	34.8	3.6	34.8	23.9	43.6	32.9	37.1
MCH [pg]	81	10.7	0.9	10.7	8.1	13.2	10.2	11.3
MCHC [g/L]	81	309.6	23.5	301.0	278.0	392.0	291.0	327.0
Platelets [G/L]	81	450.1	313.3	415.0	119.0	2439.0	319.0	484.0
Neutrophils [%]	81	51.2	9.5	52.0	28.0	78.0	44.0	58.0
Lymphocytes [%]	81	30.7	8.3	29.0	9.0	52.0	25.0	35.0
Monocytes [%]	81	1.2	1.1	1.0	0.0	6.0	1.0	1.0
Eosinophils [%]	81	16.0	7.5	15.0	0.0	39.0	11.0	20.0
Basophils [%]	81	0.0	0.2	0.0	0.0	1.0	0.0	0.0
Neutrophils [G/L]	81	6.7	2.4	6.3	3.1	17.9	5.3	8.1
Lymphocytes [G/L]	81	4.0	1.6	3.6	1.6	9.0	2.9	4.9
Monocytes [G/L]	81	0.2	0.2	0.1	0.0	0.9	0.1	0.2
Eosinophils [G/L]	81	2.1	1.3	1.9	0.0	8.9	1.3	2.5
Basophils [G/L]	81	0.0	0.0	0.0	0.0	0.2	0.0	0.0
NLR	81	1.87	1.00	1.81	0.62	8.67	1.20	2.21
PLR	81	129.2	133.1	106.8	26.3	1183.4	76.2	150.8
LMR	63	24.7	10.9	25.0	4.0	52.0	17.5	30.0
Iron [µmol/L]	81	26.1	6.8	25.6	7.0	47.6	23.3	30.3

SD: standard deviation, Min: minimum, Max: maximum, LQ: lower quartile, UQ: upper quartile. WBC: White blood cell, RBC: Red blood cell, Hb: Hemoglobin, HCT: Hematocrit, MCV: Mean corpuscular volume, MCH: Mean corpuscular hemoglobin, MCHC: Mean corpuscular hemoglobin concentration, NLR: Neutrophil-to-lymphocyte ratio, PLR: Platelet-to-lymphocyte ratio, LMR: Lymphocyte-to-monocyte ratio.

Supplementary Table 3:

Descriptive results of the findings for group “Y” (young; animals at 1 year of age) for the hematological parameters and for serum iron.

	n=	Mean	SD	Median	Min	Max	LQ	UQ
WBC [G/L]	11	13.5	3.2	12.4	9.7	20.4	11.6	16.0
RBC [T/L]	11	12.5	1.2	12.5	11.0	14.6	11.3	13.6
Hb [g/L]	11	129.5	8.9	128.0	118.0	149.0	123.0	135.0
HCT [L/L]	11	0.40	0.04	0.40	0.35	0.48	0.37	0.41
MCV [fL]	11	31.9	2.5	32.4	27.6	36.9	30.1	33.5
MCH [pg]	11	10.4	0.7	10.7	8.9	11.3	9.9	10.9
MCHC [g/L]	11	327.3	12.8	324.0	307.0	348.0	319.0	339.0
Platelets [G/L]	11	445.1	221.5	415.0	199.0	970.0	245.0	565.0
Neutrophils [%]	11	52.5	7.1	55.0	44.0	64.0	44.0	58.0
Lymphocytes [%]	11	35.0	5.9	35.0	26.0	44.0	31.0	40.0
Monocytes [%]	11	1.5	1.2	1.0	0.0	4.0	1.0	2.0
Eosinophils [%]	11	9.8	4.6	8.0	3.0	17.0	6.0	14.0
Basophils [%]	11	0.0	0.0	0.0	0.0	0.0	0.0	0.0
Neutrophils [G/L]	11	7.1	1.9	6.4	5.1	9.9	5.3	9.2
Lymphocytes [G/L]	11	4.8	1.7	4.3	3.0	9.0	3.4	5.5
Monocytes [G/L]	11	0.2	0.2	0.1	0.0	0.8	0.1	0.3
Eosinophils [G/L]	11	1.3	0.5	1.4	0.5	2.0	0.7	1.7
Basophils [G/L]	11	0.0	0.0	0.0	0.0	0.0	0.0	0.0
NLR	11	1.57	0.44	1.57	1.00	2.29	1.10	1.90
PLR	11	98.9	48.0	91.5	36.5	163.9	59.8	150.8
LMR	9	24.4	9.7	26.0	10.3	35.0	18.5	34.0
Iron [µmol/L]	11	22.9	8.9	23.6	7.3	39.0	14.9	28.3

SD: standard deviation, Min: minimum, Max: maximum, LQ: lower quartile, UQ: upper quartile. WBC: White blood cell, RBC: Red blood cell, Hb: Hemoglobin, HCT: Hematocrit, MCV: Mean corpuscular volume, MCH: Mean corpuscular hemoglobin, MCHC: Mean corpuscular hemoglobin concentration, NLR: Neutrophil-to-lymphocyte ratio, PLR: Platelet-to-lymphocyte ratio, LMR: Lymphocyte-to-monocyte ratio.

Supplementary Table 4:

Descriptive results of the findings for group “M” (mature; animals at ≥ 4 years of age) for the hematological parameters and for serum iron.

	n=	Mean	SD	Median	Min	Max	LQ	UQ
WBC [G/L]	11	12.3	2.8	11.1	8.8	17.1	10.0	15.0
RBC [T/L]	11	12.9	1.2	12.9	10.7	14.2	11.9	14.0
Hb [g/L]	11	136.8	11.1	136.0	117.0	153.0	128.0	146.0
HCT [L/L]	11	0.44	0.05	0.43	0.37	0.52	0.38	0.48
MCV [fL]	11	34.0	3.2	33.2	29.6	40.1	31.7	36.9
MCH [pg]	11	10.6	0.6	10.6	9.5	11.8	10.3	11.1
MCHC [g/L]	11	313.9	26.4	302.0	286.0	349.0	288.0	339.0
Platelets [G/L]	11	302.7	101.9	333.0	146.0	425.0	197.0	389.0
Neutrophils [%]	11	53.5	7.6	54.0	37.0	62.0	48.0	61.0
Lymphocytes [%]	11	29.4	7.0	30.0	21.0	48.0	25.0	30.0
Monocytes [%]	11	1.4	0.8	1.0	0.0	3.0	1.0	2.0
Eosinophils [%]	11	15.4	4.5	16.0	7.0	24.0	12.0	18.0
Basophils [%]	11	0.0	0.0	0.0	0.0	0.0	0.0	0.0
Neutrophils [G/L]	11	6.6	1.8	6.2	4.5	9.8	5.4	8.7
Lymphocytes [G/L]	11	3.7	1.5	3.3	2.6	8.2	2.9	3.6
Monocytes [G/L]	11	0.2	0.1	0.1	0.0	0.5	0.1	0.3
Eosinophils [G/L]	11	1.9	0.6	1.9	0.7	3.1	1.4	2.1
Basophils [G/L]	11	0.0	0.0	0.0	0.0	0.0	0.0	0.0
NLR	11	1.94	0.57	1.78	0.77	2.90	1.69	2.44
PLR	11	93.5	44.6	105.3	27.9	161.0	55.0	115.6
LMR	10	22.2	7.9	25.5	10.5	30.0	15.0	30.0
Iron [µmol/L]	11	26.4	5.5	27.8	15.2	33.3	23.3	30.1

SD: standard deviation, Min: minimum, Max: maximum, LQ: lower quartile, UQ: upper quartile. WBC: White blood cell, RBC: Red blood cell, Hb: Hemoglobin, HCT: Hematocrit, MCV: Mean corpuscular volume, MCH: Mean corpuscular hemoglobin, MCHC: Mean corpuscular hemoglobin concentration, NLR: Neutrophil-to-lymphocyte ratio, PLR: Platelet-to-lymphocyte ratio, LMR: Lymphocyte-to-monocyte ratio.

Supplementary Table 5:

Descriptive results of the findings for animals from groups “Y” (young; animals at 1 year of age) and “M” (mature; animals at ≥ 4 years of age) with “IL” (iron low; serum iron < 26.0 µmol/L) for the hematological parameters and for serum iron.

	n=	Mean	SD	Median	Min	Max	LQ	UQ
WBC [G/L]	13	13.4	3.0	12.4	10.0	20.4	11.1	14.4
RBC [T/L]	13	12.9	1.1	12.7	11.3	14.6	12.1	13.8
Hb [g/L]	13	132.1	9.7	132.0	117.0	149.0	123.0	138.0
HCT [L/L]	13	0.41	0.04	0.40	0.35	0.48	0.38	0.43
MCV [fL]	13	31.5	1.8	31.9	27.6	33.6	30.4	33.0
MCH [pg]	13	10.3	0.7	10.2	8.9	11.1	9.9	10.9
MCHC [g/L]	13	326.2	18.0	327.0	286.0	349.0	321.0	339.0
Platelets [G/L]	13	398.8	215.7	369.0	176.0	970.0	245.0	425.0
Neutrophils [%]	13	54.0	7.3	56.0	44.0	64.0	47.0	59.0
Lymphocytes [%]	13	33.3	6.3	31.0	25.0	44.0	30.0	37.0
Monocytes [%]	13	1.3	1.2	1.0	0.0	4.0	1.0	2.0
Eosinophils [%]	13	10.5	4.8	11.0	3.0	18.0	7.0	14.0
Basophils [%]	13	0.0	0.0	0.0	0.0	0.0	0.0	0.0
Neutrophils [G/L]	13	7.2	1.8	6.6	5.3	9.9	5.5	9.0
Lymphocytes [G/L]	13	4.5	1.7	4.1	2.7	9.0	3.3	5.2
Monocytes [G/L]	13	0.2	0.2	0.1	0.0	0.8	0.1	0.2
Eosinophils [G/L]	13	1.3	0.5	1.5	0.5	2.0	0.7	1.7
Basophils [G/L]	13	0.0	0.0	0.0	0.0	0.0	0.0	0.0
NLR	13	1.70	0.48	1.70	1.00	2.44	1.34	2.07
PLR	13	92.0	42.9	91.5	36.5	163.9	55.5	115.6
LMR	10	23.6	8.2	25.5	10.3	35.0	18.5	30.0
Iron [µmol/L]	13	20.1	5.7	23.3	7.3	25.6	15.2	24.7

SD: standard deviation, Min: minimum, Max: maximum, LQ: lower quartile, UQ: upper quartile. WBC: White blood cell, RBC: Red blood cell, Hb: Hemoglobin, HCT: Hematocrit, MCV: Mean corpuscular volume, MCH: Mean corpuscular hemoglobin, MCHC: Mean corpuscular hemoglobin concentration, NLR: Neutrophil-to-lymphocyte ratio, PLR: Platelet-to-lymphocyte ratio, LMR: Lymphocyte-to-monocyte ratio.

Supplementary Table 6:

Descriptive results of the findings for animals from groups "Y" (young; animals at 1 year of age) and "M" (mature; animals at ≥ 4 years of age) with "IH" (iron high; serum iron ≥ 26.0 µmol/L) for the hematological parameters and for serum iron.

	n=	Mean	SD	Median	Min	Max	LQ	UQ
WBC [G/L]	9	12.3	3.1	11.6	8.8	17.1	9.7	15.0
RBC [T/L]	9	12.4	1.3	12.6	10.7	14.0	11.1	13.8
Hb [g/L]	9	134.8	12.0	130.0	118.0	153.0	127.0	146.0
HCT [L/L]	9	0.44	0.06	0.43	0.37	0.52	0.38	0.48
MCV [fL]	9	35.1	3.1	36.0	29.6	40.1	33.4	36.9
MCH [pg]	9	10.9	0.5	10.7	10.3	11.8	10.6	11.2
MCHC [g/L]	9	312.4	24.2	307.0	287.0	349.0	292.0	333.0
Platelets [G/L]	9	337.9	126.3	333.0	146.0	512.0	229.0	425.0
Neutrophils [%]	9	51.7	7.3	51.0	37.0	61.0	48.0	55.0
Lymphocytes [%]	9	30.6	7.9	30.0	21.0	48.0	26.0	34.0
Monocytes [%]	9	1.6	0.7	1.0	1.0	3.0	1.0	2.0
Eosinophils [%]	9	15.7	4.6	16.0	8.0	24.0	14.0	17.0
Basophils [%]	9	0.0	0.0	0.0	0.0	0.0	0.0	0.0
Neutrophils [G/L]	9	6.3	1.9	6.2	4.5	9.8	5.1	6.4
Lymphocytes [G/L]	9	3.8	1.7	3.4	2.6	8.2	3.0	3.5
Monocytes [G/L]	9	0.2	0.1	0.1	0.1	0.5	0.1	0.3
Eosinophils [G/L]	9	1.9	0.7	2.0	0.8	3.1	1.4	2.1
Basophils [G/L]	9	0.0	0.0	0.0	0.0	0.0	0.0	0.0
NLR	9	1.82	0.62	1.70	0.77	2.90	1.57	2.12
PLR	9	102.2	50.6	108.1	27.9	163.1	66.4	150.8
LMR	9	22.8	9.5	26.0	10.5	35.0	15.0	30.0
Iron [µmol/L]	9	31.2	3.5	30.1	27.8	39.0	28.8	32.4

SD: standard deviation, Min: minimum, Max: maximum, LQ: lower quartile, UQ: upper quartile. WBC: White blood cell, RBC: Red blood cell, Hb: Hemoglobin, HCT: Hematocrit, MCV: Mean corpuscular volume, MCH: Mean corpuscular hemoglobin, MCHC: Mean corpuscular hemoglobin concentration, NLR: Neutrophil-to-lymphocyte ratio, PLR: Platelet-to-lymphocyte ratio, LMR: Lymphocyte-to-monocyte ratio.

Supplementary Table 7:

Descriptive results of the findings for animals from group “YIL” (young iron low; animals at 1 year of age; serum iron < 26.0 µmol/L) for the hematological parameters and for serum iron.

	n=	Mean	SD	Median	Min	Max	LQ	UQ
WBC [G/L]	8	14.7	2.9	14.2	11.7	20.4	12.2	16.4
RBC [T/L]	8	13.0	1.1	12.9	11.3	14.6	12.3	13.7
Hb [g/L]	8	131.6	9.3	131.5	122.0	149.0	123.0	136.5
HCT [L/L]	8	0.40	0.04	0.40	0.35	0.48	0.38	0.42
MCV [fL]	8	31.0	2.0	31.2	27.6	33.6	29.7	32.5
MCH [pg]	8	10.2	0.7	10.2	8.9	10.9	9.9	10.9
MCHC [g/L]	8	329.0	11.4	325.5	313.0	348.0	322.0	338.0
Platelets [G/L]	8	458.0	249.4	392.0	199.0	970.0	277.5	578.0
Neutrophils [%]	8	52.3	8.3	52.5	44.0	64.0	44.0	58.5
Lymphocytes [%]	8	36.3	6.1	36.0	28.0	44.0	31.0	42.0
Monocytes [%]	8	1.6	1.4	1.5	0.0	4.0	0.5	2.5
Eosinophils [%]	8	8.6	4.3	7.5	3.0	15.0	5.5	12.5
Basophils [%]	8	0.0	0.0	0.0	0.0	0.0	0.0	0.0
Neutrophils [G/L]	8	7.7	2.0	8.5	5.3	9.9	5.5	9.2
Lymphocytes [G/L]	8	5.3	1.6	5.0	4.0	9.0	4.2	5.7
Monocytes [G/L]	8	0.3	0.3	0.2	0.0	0.8	0.1	0.4
Eosinophils [G/L]	8	1.2	0.5	1.4	0.5	1.8	0.7	1.7
Basophils [G/L]	8	0.0	0.0	0.0	0.0	0.0	0.0	0.0
NLR	8	1.51	0.48	1.46	1.00	2.29	1.05	1.89
PLR	8	88.5	45.2	78.1	36.5	163.9	53.0	122.7
LMR	6	20.8	9.7	20.3	10.3	35.0	11.0	28.0
Iron [µmol/L]	8	19.1	6.5	21.4	7.3	25.6	14.5	24.2

SD: standard deviation, Min: minimum, Max: maximum, LQ: lower quartile, UQ: upper quartile. WBC: White blood cell, RBC: Red blood cell, Hb: Hemoglobin, HCT: Hematocrit, MCV: Mean corpuscular volume, MCH: Mean corpuscular hemoglobin, MCHC: Mean corpuscular hemoglobin concentration, NLR: Neutrophil-to-lymphocyte ratio, PLR: Platelet-to-lymphocyte ratio, LMR: Lymphocyte-to-monocyte ratio.

Supplementary Table 8:

Descriptive results of the findings for animals from group “YIH” (young iron high; animals at 1 year of age; serum iron ≥ 26.0 µmol/L) for the hematological parameters and for serum iron.

	n=	Mean	SD	Median	Min	Max	LQ	UQ
WBC [G/L]	3	10.5	1.0	10.1	9.7	11.6	9.7	11.6
RBC [T/L]	3	11.2	0.2	11.1	11.0	11.5	11.0	11.5
Hb [g/L]	3	124.0	5.3	126.0	118.0	128.0	118.0	128.0
HCT [L/L]	3	0.39	0.02	0.38	0.37	0.41	0.37	0.41
MCV [fL]	3	34.4	2.2	33.5	32.7	36.9	32.7	36.9
MCH [pg]	3	11.1	0.3	11.2	10.7	11.3	10.7	11.3
MCHC [g/L]	3	322.7	17.8	319.0	307.0	342.0	307.0	342.0
Platelets [G/L]	3	410.7	158.5	492.0	228.0	512.0	228.0	512.0
Neutrophils [%]	3	53.3	2.9	55.0	50.0	55.0	50.0	55.0
Lymphocytes [%]	3	31.7	4.9	34.0	26.0	35.0	26.0	35.0
Monocytes [%]	3	1.0	0.0	1.0	1.0	1.0	1.0	1.0
Eosinophils [%]	3	13.0	4.6	14.0	8.0	17.0	8.0	17.0
Basophils [%]	3	0.0	0.0	0.0	0.0	0.0	0.0	0.0
Neutrophils [G/L]	3	5.6	0.7	5.3	5.1	6.4	5.1	6.4
Lymphocytes [G/L]	3	3.3	0.2	3.4	3.0	3.4	3.0	3.4
Monocytes [G/L]	3	0.1	0.0	0.1	0.1	0.1	0.1	0.1
Eosinophils [G/L]	3	1.4	0.6	1.4	0.8	2.0	0.8	2.0
Basophils [G/L]	3	0.0	0.0	0.0	0.0	0.0	0.0	0.0
NLR	3	1.72	0.35	1.57	1.47	2.12	1.47	2.12
PLR	3	126.8	52.7	150.8	66.4	163.1	66.4	163.1
LMR	3	31.7	4.9	34.0	26.0	35.0	26.0	35.0
Iron [µmol/L]	3	33.1	5.4	32.1	28.3	39.0	28.3	39.0

SD: standard deviation, Min: minimum, Max: maximum, LQ: lower quartile, UQ: upper quartile. WBC: White blood cell, RBC: Red blood cell, Hb: Hemoglobin, HCT: Hematocrit, MCV: Mean corpuscular volume, MCH: Mean corpuscular hemoglobin, MCHC: Mean corpuscular hemoglobin concentration, NLR: Neutrophil-to-lymphocyte ratio, PLR: Platelet-to-lymphocyte ratio, LMR: Lymphocyte-to-monocyte ratio.

Supplementary Table 9:

Descriptive results of the findings for animals from group “MIL” (mature iron low; animals at ≥ 4 years of age; serum iron < 26.0 μmol/L) for the hematological parameters and for serum iron.

	n=	Mean	SD	Median	Min	Max	LQ	UQ
WBC [G/L]	5	11.3	1.7	10.7	10.0	14.2	10.4	11.1
RBC [T/L]	5	12.8	1.2	12.3	11.7	14.2	11.9	14.1
Hb [g/L]	5	132.8	11.3	132.0	117.0	146.0	128.0	141.0
HCT [L/L]	5	0.41	0.04	0.41	0.38	0.47	0.38	0.43
MCV [fL]	5	32.3	1.2	33.0	30.4	33.2	31.7	33.0
MCH [pg]	5	10.4	0.7	10.3	9.5	11.1	10.0	11.0
MCHC [g/L]	5	321.8	26.6	333.0	286.0	349.0	302.0	339.0
Platelets [G/L]	5	304.2	112.0	338.0	176.0	425.0	197.0	385.0
Neutrophils [%]	5	56.8	4.7	56.0	51.0	62.0	54.0	61.0
Lymphocytes [%]	5	28.6	3.0	30.0	25.0	32.0	26.0	30.0
Monocytes [%]	5	0.8	0.4	1.0	0.0	1.0	1.0	1.0
Eosinophils [%]	5	13.4	4.2	14.0	7.0	18.0	12.0	16.0
Basophils [%]	5	0.0	0.0	0.0	0.0	0.0	0.0	0.0
Neutrophils [G/L]	5	6.4	1.3	5.8	5.4	8.7	5.7	6.6
Lymphocytes [G/L]	5	3.2	0.3	3.2	2.7	3.6	3.2	3.3
Monocytes [G/L]	5	0.1	0.1	0.1	0.0	0.1	0.1	0.1
Eosinophils [G/L]	5	1.5	0.5	1.7	0.7	2.0	1.4	1.7
Basophils [G/L]	5	0.0	0.0	0.0	0.0	0.0	0.0	0.0
NLR	5	2.01	0.32	2.07	1.69	2.44	1.70	2.15
PLR	5	97.7	43.4	105.3	55.0	157.2	55.5	115.6
LMR	4	27.8	2.6	28.0	25.0	30.0	25.5	30.0
Iron [μmol/L]	5	21.7	4.4	23.3	15.2	25.6	19.5	25.1

SD: standard deviation, Min: minimum, Max: maximum, LQ: lower quartile, UQ: upper quartile. WBC: White blood cell, RBC: Red blood cell, Hb: Hemoglobin, HCT: Hematocrit, MCV: Mean corpuscular volume, MCH: Mean corpuscular hemoglobin, MCHC: Mean corpuscular hemoglobin concentration, NLR: Neutrophil-to-lymphocyte ratio, PLR: Platelet-to-lymphocyte ratio, LMR: Lymphocyte-to-monocyte ratio.

Supplementary Table 10:

Descriptive results of the findings for animals from group “MIH” (mature iron high; animals at ≥ 4 years of age; serum iron ≥ 26.0 µmol/L) for the hematological parameters and for serum iron.

	n=	Mean	SD	Median	Min	Max	LQ	UQ
WBC [G/L]	6	13.2	3.4	14.0	8.8	17.1	9.6	16.0
RBC [T/L]	6	13.0	1.2	13.3	10.7	14.0	12.6	13.9
Hb [g/L]	6	140.2	10.7	141.0	127.0	153.0	130.0	149.0
HCT [L/L]	6	0.46	0.05	0.47	0.37	0.52	0.43	0.50
MCV [fL]	6	35.5	3.6	36.5	29.6	40.1	33.4	37.1
MCH [pg]	6	10.8	0.5	10.7	10.3	11.8	10.5	11.1
MCHC [g/L]	6	307.3	26.7	293.5	287.0	349.0	288.0	333.0
Platelets [G/L]	6	301.5	103.5	310.0	146.0	425.0	229.0	389.0
Neutrophils [%]	6	50.8	8.9	49.5	37.0	61.0	48.0	60.0
Lymphocytes [%]	6	30.0	9.5	28.5	21.0	48.0	24.0	30.0
Monocytes [%]	6	1.8	0.8	2.0	1.0	3.0	1.0	2.0
Eosinophils [%]	6	17.0	4.3	16.0	12.0	24.0	14.0	20.0
Basophils [%]	6	0.0	0.0	0.0	0.0	0.0	0.0	0.0
Neutrophils [G/L]	6	6.7	2.2	6.3	4.5	9.8	4.6	9.0
Lymphocytes [G/L]	6	4.0	2.1	3.4	2.6	8.2	2.9	3.6
Monocytes [G/L]	6	0.3	0.2	0.2	0.1	0.5	0.1	0.3
Eosinophils [G/L]	6	2.2	0.6	2.1	1.4	3.1	1.9	2.6
Basophils [G/L]	6	0.0	0.0	0.0	0.0	0.0	0.0	0.0
NLR	6	1.88	0.75	1.74	0.77	2.90	1.60	2.50
PLR	6	90.0	49.4	96.7	27.9	161.0	41.9	115.6
LMR	6	18.4	8.1	15.5	10.5	30.0	12.0	27.0
Iron [µmol/L]	6	30.3	2.2	29.7	27.8	33.3	28.8	32.4

SD: standard deviation, Min: minimum, Max: maximum, LQ: lower quartile, UQ: upper quartile. WBC: White blood cell, RBC: Red blood cell, Hb: Hemoglobin, HCT: Hematocrit, MCV: Mean corpuscular volume, MCH: Mean corpuscular hemoglobin, MCHC: Mean corpuscular hemoglobin concentration, NLR: Neutrophil-to-lymphocyte ratio, PLR: Platelet-to-lymphocyte ratio, LMR: Lymphocyte-to-monocyte ratio.
